# Development of a TaqMan qPCR for the Simultaneous Detection of the TuMV and BBWV2 Viruses Responsible for the Viral Disease in *Pseudostellaria heterophylla*

**DOI:** 10.3390/microorganisms12122663

**Published:** 2024-12-22

**Authors:** Li Gu, Chensi Liu, Shuting Yao, Jiaxin Wu, Lianghong Wang, Jing Mu, Yankun Wang, Jianming Wang, Zhongyi Zhang, Mingjie Li

**Affiliations:** 1 College of Bee Science and Biomedicine, Fujian Agriculture and Forestry University, Fuzhou 350002, China; 2 Key Laboratory of Ministry of Education for Genetics, Breeding and Multiple Utilization of Crops, Fujian Agriculture and Forestry University, Fuzhou 350002, China; 3 State Key Laboratory for Quality Ensurance and Sustainable Use of Dao-di Herbs, National Resource Center for Chinese Meteria Medica, China Academy of Chinese Medical Sciences, Beijing 100700, China

**Keywords:** *Pseudostellaria heterophylla*, infectious clone, TaqMan qPCR, virus detection, TuMV, BBWV2

## Abstract

*Pseudostellaria heterophylla* (Miq.) Pax, a highly valued Chinese medicinal plant, is experiencing a notable decline in yield and quality due to viral diseases, particularly caused those by TuMV and BBWV2. Currently, the absence of a quantitative detection method for these viruses in *P. heterophylla* impedes the accurate diagnosis. The development of an accurate quantitative detection method is thus essential for effectively managing viral diseases in this plant. In this study, singleplex and duplex TaqMan qPCR were developed for the detection of the two viruses, based on two viral cloning vectors. Concurrently, the levels of both viruses were examined in the main produced regions of *P. heterophylla*. Furthermore, the levels of BBWV2 were examined during its infection of *P. heterophylla*. The optimal singleplex qPCR employed 0.1 μM of hydrolysis probe and 0.1 μM of primer for TuMV, while 0.2 μM of hydrolysis probe and 0.1 μM of primer were utilised for BBWV2. In contrast, the duplex qPCR employed the use of 0.1 μM of the upstream/downstream primer from each primer pair, with 0.2 μM of the respective hydrolysis probes. The 95% limit of detection (LOD) for singleplex qPCR was 734 copies for TuMV and 20 copies for BBWV2, while the 95% LOD for duplex qPCR was 945 copies for TuMV and 47 copies for BBWV2. Furthermore, the intra- and inter-assay coefficients of variation were found to be less than 1.2% for both singleplex and duplex qPCR. Of the *P. heterophylla* sampled 60 sites, 96% were found to be infected by one of two viruses. The levels of BBWV2 in *N. benthamiana* and *P. heterophylla* demonstrated an initial increase, followed by a subsequent decrease. The TaqMan qPCR methods provide a technical foundation for the monitoring of virus infections in *P. heterophylla*.

## 1. Introduction

*Pseudostellaria heterophylla* (*P. heterophylla*) is a perennial herb belonging to the Caryophyllaceae family. It is a commonly utilised Chinese herbal medicine in China. In recent years, the widespread application of *P. heterophylla* in traditional Chinese medicine has led to a notable increase in demand for this herb, accompanied by a corresponding growth in its cultivation area in China. However, the production of *P. heterophylla* is significantly hindered by viral disease, with an incidence reaching 60% to 90% in the field, and even 100% in severe cases [1,2]. The infection of *P. heterophylla* by viruses resulted in notable changes in growth and development, as evidenced by a range of observable symptoms, including dwarf plants, mottled and curled leaves, and a decline in the number and size of tuberous roots [2]. Furthermore, *P. heterophylla* utilises tuberous roots as reproductive materials in the field of production. As the number of reproductive generations increases, the occurrence of viral infections in *P. heterophylla* also rises, resulting in a reduction in the quality and yield of *P. heterophylla* [2,3]. The viral disease is currently a significant constraint on the advancement of the entire industry of *P. heterophylla*. It is therefore crucial to ascertain the source of infection of *P. heterophylla* viral disease and to propose the corresponding control measures.

The identification of pathogens associated with the *P. heterophylla* viral disease was previously conducted for many years in China. The causal agent of *P. heterophylla* viral disease was identified and named the Taizishen mosaic virus (TaMV) by the early 1980s [4]. Subsequent studies have demonstrated the coexistence of additional viruses within infected *P. heterophylla*. These include turnip mosaic virus (TuMV), cucumber mosaic virus (CMV), broad bean wilt virus 2 (BBWV2), and tobacco mosaic virus (TMV) [5]. A variety of techniques have been employed for the detection of *P. heterophylla* viruses in samples collected from disparate cultivation areas in China. For example, CMV and TMV have been identified in *P. heterophylla* samples using ELISA [6,7]. Moreover, the conserved nature of the coat protein (CP) genes from TuMV or BBWV2 across various plants has enabled the utilisation of RT-PCR for the detection of these two viruses in *P. heterophylla* samples [8,9]. A recent study employed RT-PCR methods to analyse the infection of viruses in 71 *P. heterophylla* samples collected from various planting regions across China. The results demonstrated the absence of evidence of TMV infection in the samples [10]. Furthermore, reverse transcription loop-mediated isothermal amplification (RT-LAMP) methods have been developed for the rapid detection of TuMV and BBWV viruses [11]. The application of high-throughput sequencing techniques led to the identification of five carlaviruses and one novel amalgavirus, in addition to six distinct BBWV2 subtypes in *P. heterophylla* [12]. The evidence indicates that the *P. heterophylla* viral disease is a typical complex infection involving multiple viruses, with BBWV2 and TuMV emerging as the most significant. The complexity of the viruses infecting *P. heterophylla* represents a significant challenge in the development of effective control technology for viral disease. Consequently, an accurate identification of the viruses affecting *P. heterophylla* and infection levels has become a crucial step in controlling *P. heterophylla* viral disease.

Currently, qPCR has been proven to be a highly effective method for quantifying virus levels, exhibiting notable reproductivity and the capacity for the simultaneous quantification of multiple viruses. Moreover, qPCR detection technology has been used extensively to accurately determine viral infection levels in plants and animals [13,14,15]. The fundamental prerequisite of developing a qPCR-based detection method is the initial acquisition of the complete or partially sequenced viral genome of the virus under examination. This is then transferred into a specific plasmid, which enables the calculation of the copy numbers of the detected object. The advent of high-throughput technology has facilitated the direct acquisition of virus fragments or virus-derived small RNAs (vsRNAs) within the host organism. These fragments or vsRNAs can then be assembled through the application of bioinformatics methods to effectively identify viral genome information. The viral genome in *P. heterophylla* plants has been identified by high-throughput methods [12,16,17]. In a preceding study, the complete genomic sequences for the TuMV and BBWV2 viruses in *P. heterophylla* were successfully obtained through vsRNA assembly, and the cloning plasmid of TuMV was also constructed [17]. These studies provide important information for developing a qPCR method that quantitatively detects *P. heterophylla* viral disease.

This study presents the development of a TaqMan qPCR method for the simultaneous and individual detection of the TuMV and BBWV2 viruses in *P. heterophylla*. The essential parameters in TaqMan qPCR were subjected to optimisation, and a comprehensive assessment of this method was conducted to analyse its reproducibility for detecting *P. heterophylla* viruses. Concurrently, the prevalence of the two viruses in the primary cultivation region of *P. heterophylla* was evaluated using an optimised duplex TaqMan qPCR. An infectious clone of BBWV2 derived from *P. heterophylla* was successfully constructed and employed to infect both *Nicotiana benthamiana* (*N. benthamiana*) and *P. heterophylla*. During these infections, the abundance of BBWV2 was meticulously examined through singleplex TaqMan qPCR. This study provides a useful tool for the monitoring of viral disease, the detection of virus-free seedlings, the investigation of viral infection mechanisms, and the development of precise control strategies for *P. heterophylla*.

## 2. Material and Methods

### 2.1. Planting and Management of Experimental Materials

The model plant *N. benthamiana*, which is commonly used in the study of viral diseases, and the host plant *P. heterophylla* were selected for the purpose of investigating the infection activity of *P. heterophylla* viruses. The *N. benthamiana* plants were cultivated in a controlled environment at a temperature of 26 °C with light intensities of 7000–8000 Lux, and a photoperiod of 16 h of light followed by 8 h of darkness. The virus-free tissue-cultured seedlings of *P. heterophylla* underwent an acclimation period before being transferred to nutrient-rich substrates. Thereafter, these *P. heterophylla* seedlings were transferred to a controlled environmental chamber at a temperature of 25 °C under a light intensity of 5000 to 6000 Lux with a photoperiod of 12 h of light and 12 h of dark.

To elucidate the prevalence of TuMV and BBWV2 viruses in production, *P. heterophylla* planted in Zherong County, Fujian Province, China, a major production region of *P. heterophylla*, were selected as sampling sites for virus detection. The samples were taken between May to June of 2023. The samples were promptly frozen in liquid nitrogen and stored in a −80 °C freezer until further analysis. In a preceding study [17], the pSMART-E-TuMV construct was stored in a −80 °C freezer and served as a foundation for developing a TaqMan qPCR to detect TuMV virus.

### 2.2. Construction of Clone and Infectious Plasmids of P. heterophylla BBWV2 Virus

**Figure 1:**
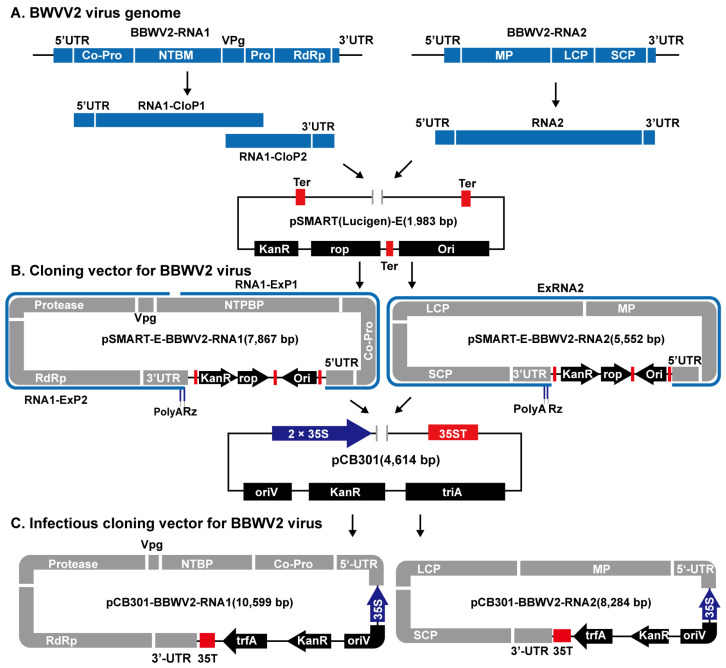
The construction process of the *P. heterophylla* BBWV2 cloning vector and infectious cloning vector. The RNA1 and RNA2 genomes from the BBWV2 virus were transferred into the pSMART (Lucigen)-E cloning vector, respectively (**A**). Subsequently, the RNA1 and RNA2 genomes of the BBWV2 virus in the pSMART-E vector were further transferred into the pCB301 expression vector, thereby forming the BBWV2 infectious clone vector (**B**,**C**).

A previous study on the viral disease of *P. heterophylla* has identified the genomic sequences of TuMV and BBWV2 viruses, as well as established the TuMV cloning and expression vector [16,17]. The BBWV2 virus comprises two single-stranded RNA molecules, RNA1 and RNA2, which were previously identified [16,17]. The two strands of BBWV2 were subjected to sequencing and were found to belong to BBWV-XC1 (ON241330 and ON241334), as identified by Li et al. [12], exhibiting a 97% and 98% similarity, respectively. The RNA1 and RNA2 genomes of the BBWV2 virus were then transferred to the clone and expression vectors, respectively, in a manner consistent with that used for the TuMV virus [17]. In brief, the RNA1 was cloned by dividing it into two sections, namely BBWV2-RNA1-P1 and BBWV2-RNA1-P2, using primers BBWV2-RNA1-ClonP1 and BBWV2-RNA1-ClonP2 from *P. heterophylla* exhibiting symptoms of viral infection in the main production regions (Table S1). Moreover, the RNA2 of the BBWV2 virus was directly cloned by BBWV-ClonRNA2 primer (Table S1). The modified vector pSMART-E [16] was linearised using pSMART-LinZ primers (Table S1). The three components, BBWV2-RNA1-ClonP1, BBWV2-RNA1-ClonP2, and the linearised pSMART-E vector, were ligated via a homologous recombination kit (Vazyme Biotech Co., Ltd., Nanjing, China) to generate a new construct named pSMART-E-BBWV2-RNA1. Similarly, the BBWV2-RNA2 was ligated with the linearised pSMART-E vector to create a new construct named pSMART-E-BBWV2-RNA2 (Figure 1).

To further construct the infectious clone vector containing RNA1 and the RNA2 genome of BBWV2, the RNA1 of BBWV2 was cloned from the pSMART-BBWV2-RNA1 vector in two segments, labelled as BBWV2-RNA1-ExP1 and BBWV2-RNA1-ExP2, using specific primers (Table S1). The polyA and RZ (self-cleaving ribozyme) sequences (Table S1) were incorporated behind the 3’ ends of BBWV2-RNA1-ExP2. Concurrently, the pCB301-LinZ primers were employed to linearise pCB301, an expression vector [18]. A homologous recombination kit (Vazyme Biotech Co., Ltd., Nanjing, China) was used to ligate BBWV2-RNA1-ExP1, BBWV2-RNA1-ExP2, and the linearised pCB301 vector, thereby forming the pCB301-BBWV2-RNA1 expression vector. The BBWV2 RNA2 genome was directly cloned from the pSMART-BBWV2-RNA2 vector using specific primers (Table S1), with the addition of polyA and RZ sequences (Table S1) in the 3’ ends of primer. Subsequently, the BBWV-RNA2 and the linearised pCB301 vector were ligated to form the pCB301-BBWV2-RNA2 expression vector, employing the same methodology as described above (Figure 1).

### 2.3. Design of TaqMan qPCR Primers and Hydrolysis Probes for P. heterophylla TuMV and BBWV2 Viruses

In between the RNA 1 and RNA 2 strands from the BBWV2 virus, RNA2 was selected as the object of qPCR detection. Furthermore, TuMV is a single-stranded RNA molecule that serves as the direct subject of qPCR detection. The conserved regions of BBWV2-RNA2 and TuMV were identified through the alignment of a variety of virus sequences derived from diverse plants, employing the Clustal Omega v1.2.4 software (https://www.ebi.ac.uk/jdispatcher/msa/clustalo, accessed on 12 June 2023). The PrimerQuest™ Tool online software (https://sg.idtdna.com/pages/tools/primerquest, accessed on 12 June 2023) was used for the qPCR TaqMan primer design following conventional guidelines. This was carried out in order to develop the optimal primers and hydrolysis probes for both viruses, with a particular focus on their conserved regions, as identified above by Clustal Omega software. To improve the efficiency of the detection process, the two virus hydrolysis probes were labelled with distinct fluorescent residues exhibiting different wavelengths. The BBWV2-RNA2 probe was labelled with a HEX fluorescent residue at its 5’ end and a BHQ2 quencher at its 3’ end, whereas the TuMV probe was labelled with a FAM fluorophore at its 5’ end and a BHQ1 quencher at its 3’ end (Table S1).

### 2.4. Optimisation of the Singleplex and Duplex TaqMan qPCR Assays

The *P. heterophylla* pSMART-BBWV2-RNA2 and pSMART-TuMV plasmids were employed as templates for the singleplex and duplex viral TaqMan qPCR assays, with free virus ddH_2_O serving as a negative control. The TaqMan qPCR were set up in a total volume of 20 μL comprising 0.2 μM upstream and downstream primers, 0.1 μM probe, and 10 μL of 2 × AceQ Universal U + Probe Master Mix V2 (Vazyme Biotech Co., Ltd., Nanjing, China), 1 μL template, made up to 20 μL with ddH_2_O. The reaction was performed at 37 °C for 2 min, followed by 95 °C for 5 min, 95 °C for 10 s, and then annealed at different optimal temperatures for 30 s each for 45 cycles. The annealing temperatures were set at 56 °C, 58 °C, 60 °C, 62 °C, and 64 °C, respectively. At the same time, based on the above qPCR, the upstream/downstream primers and the hydrolysis probe for each virus were set to range from 0.1 to 0.4 μM and from 0.05 to 0.25 μM, respectively. The optimal concentration of hydrolysis probes and primers in the qPCR for the detection of singleplex and duplex viruses was determined based on several criteria, including the quantification cycle (Cq) value, fluorescence increment, amplification efficiency, and platform period of qPCR under different conditions. All qPCR assays were performed on the BioRad CFX96 qPCR system (Bio-Rad Laboratories Co., Ltd., Hercules, CA, USA).

### 2.5. Linear Equation of Concentration of Virus Plasmid with Cq Value

The pSMART-BBWV2-RNA2 and pSMART-TuMV plasmids were employed as standard plasmids in TaqMan qPCR. The total molecular weight of the two plasmids was calculated, and the plasmid copy number was determined using the following equation: plasmid copy number (copies/μL) = (6.02 × 10^23^) × (plasmid concentration ng/μL × 10⁻⁹)/(plasmid length × 660). To provide a realistic estimate of the virus levels in plants, the copy number of these two viruses was calculated using their respective cloning plasmids including the full viral genome. For determining the initial lower limit detection of the TaqMan qPCR, two standard plasmids were diluted in a 10-fold gradient from 10^5^ to 10^0^ copies/μL. Each concentration was tested in three replicates using the optimised TaqMan qPCR. Specifically, the lowest concentration of the standard plasmid that yielded a Cq value of ≤35 was confirmed to be positive. Based on these preliminary results, an optimal detection range was identified for further analysis. To construct accurate standard curves, the selected concentrations of the standard plasmids within this range were re-tested using both singleplex and multiplex PCR. The results were then used to plot detection curves, with Cq values on the *y*-axis and the logarithm of the initial copy numbers on the *x*-axis.

### 2.6. Sensitivity and Reproducibility Test of Singleplex and Duplex qPCR Assays

For the rigorous validation of the limit of detection at a 95% confidence level (95% LOD) for TaqMan qPCR, each dilution from 10^5^ to 10^0^ copies/μL was subjected to qPCR tests, with eight replicates per dilution. The 95% LOD was determined using probit regression analysis. Moreover, 10-fold serial dilutions of two plasmid standards, ranging from 10^4^ to 10^6^ copies/μL, were employed as templates to assess the intra-group and inter-group reproducibility of singleplex and duplex viruses using TaqMan qPCR. The plasmid standards of each dilution gradient were detected five times in parallel using optimised TaqMan qPCR in order to calculate the intra-assay coefficient of variation. Furthermore, the plasmid standards of each dilution gradient were detected in three batches at different time points in order to calculate the inter-assay coefficient of variation. The reproducibility of the qPCR was evaluated through the calculation of the intra- and inter-assay coefficient of variation.

### 2.7. Detection of TuMV and BBWV2 in the Main Production Areas of P. heterophylla

In the main cultivation area of *P. heterophylla*, a total of 60 sampling sites were established, with six plants sampled at each site. Three plants were randomly selected from each site for virus detection. The total RNA was extracted from the samples of *P. heterophylla* using the Trizol method, as described in a previous study [17]. Subsequently, cDNA 1st strand synthesis was conducted utilising the HiScript II 1st Strand cDNA Synthesis Kit (Vazyme Biotech Co., Ltd., Nanjing, China) and random primers as guided sequences. A duplex virus TaqMan qPCR was employed to quantify the levels of the two viruses in different samples. 

### 2.8. Investigation of BBWV2 Virus Levels During Its Infection Against N. benthamiana and P. heterophylla

The pCB301-BBWV2-RNA1 and pCB301-BBWV2-RNA2 plasmids were transferred into *Agrobacterium tumefaciens* (*A. tumefaciens*) GV3101 via the freeze–thaw method. Subsequently, the positive GV3101 clones were cultivated continuously in LB liquid medium. After being harvested at 4000 rpm for 10 min, the supernatants were discarded, leaving the pellets to be resuspended in a mixture of 10 mM MES (pH 5.6), 10 mM MgCl₂, and 200 µM acetosyringone. Furthermore, the resuspended *A. tumefaciens* GV3101 cultures, which contained equal proportions of pCB301-BBWV2-RNA1 and pCB301-BBWV2-RNA2 suspensions, were incubated at 28 °C in the dark for three hours. The needle from a 1 mL syringe was removed and used to aspirate the GV3101 suspension. This suspension was then infiltrated into the abaxial surfaces of the leaves of *N. benthamiana* plants with 4–5 leaves, as well as virus-free *P. heterophylla* seedlings with 7–8 leaves. Following the infection process, both plants were placed in darkness for 24 h, after which they were transferred to a controlled environment. Subsequently, leaf and root tissues were harvested from *N. benthamiana* and *P. heterophylla* at 3, 6, 9, 12, and 15 days after infection (DAIs). RT-PCR methods [9,17] were used to verify the infection states of BBWV2 in both plants by employing both the BBWV2-RNA1-Dect and the BBWV2-RNA2-Dect primers (Table S1). Additionally, the levels of BBWV2 in both plants were detected using the established singleplex BBWV2 TaqMan qPCR, with three biological replicates for each treatment.

## 3. Results

### 3.1. Optimisation of TaqMan qPCR for the Detection of Singleplex and Duplex Viruses

To construct a TaqMan-based qPCR method capable of detecting both viruses simultaneously or separately, the cloning vectors pSMART-E-TuMV and pSMART-E-BBWV2-RNA2 were employed as qPCR templates for the two viruses. Concurrently, the conserved regions of the two viruses were identified to obtain a candidate region for the design of primers and hydrolysis probes, effectively detect the two viruses, and avoid the effects of viral variability on the detection method. This was conducted based on the alignment results of the genomes of the two *P. heterophylla* viruses with those of other plants with close genetic relationships. The results indicated that the 9234–9405 bp region (situated within CP encoding regions) of TuMV and the 2976–3116 bp region (positioned within SCP encoding regions) of BBWV2 exhibited a relatively high level of conservation. Primers and hydrolysis probes for TuMV and BBWV2 viruses were designed in these regions. Subsequently, the associated parameters for the qPCR were optimised. With the advent of qPCR technology, PCR components can be readily prepared by incorporating optimised dNTP, MgCl_2_, and polymerase into a convenient solution. Consequently, the principal variables for consideration in the optimisation of qPCR are now the annealing temperature and the concentrations of the primers and hydrolysis probes. To ascertain the optimal levels of these three parameters in the TaqMan qPCR for the two viruses, different optimisation assays were performed. Based on the Cq values and fluorescence increments at various annealing temperatures, it was determined that 64 °C was initially identified as the optimal annealing temperature for both the singleplex and duplex TaqMan qPCR of TuMV and BBWV2 viruses, as indicated by the emergence of a single melting curve peak and the lowest Cq value (Table 1 and Table S2).

Subsequently, a comprehensive investigation was undertaken to determine the concentration of primers and hydrolysis probes in TaqMan qPCR. The findings revealed that in the singleplex TaqMan qPCR for the BBWV2, with the upstream and downstream primer of 0.1 μM and hydrolysis probe of 0.2 μM, the melting curve exhibited the lowest Cq value (Table S3). Concerning TuMV, the optimal upstream and downstream primer and hydrolysis probe concentrations were identified as 0.1 μM and 0.1 μM, respectively, exhibiting a single melting curve peak and the lowest Cq value (Table S3). Simultaneously, an optimisation of the combination of primers and hydrolysis probes from the BBWV2 and TuMV viruses for use in a duplex TaqMan qPCR was performed, to facilitate the simultaneous detection of these two viruses. The results demonstrated that in a duplex TaqMan PCR assay, the combination of two sets of 0.1 μM upstream and downstream primers with two 0.2 μM hydrolysis probes led to the generation of relatively low Cq values in both TuMV and BBWV2 (Table 2). Moreover, the Cq values of BBWV2 under this combination of primer and hydrolysis probe concentration exhibited the closest similarity to those of TuMV (Table 2). By employing these optimisation assays, the optimal annealing temperature and the most suitable primer and hydrolysis probe concentration for both the singleplex and duplex TaqMan qPCR of the two viruses were ultimately determined.

### 3.2. Establishment of Standard Curve Between Cq and Virus Concentration

**Figure 2:**
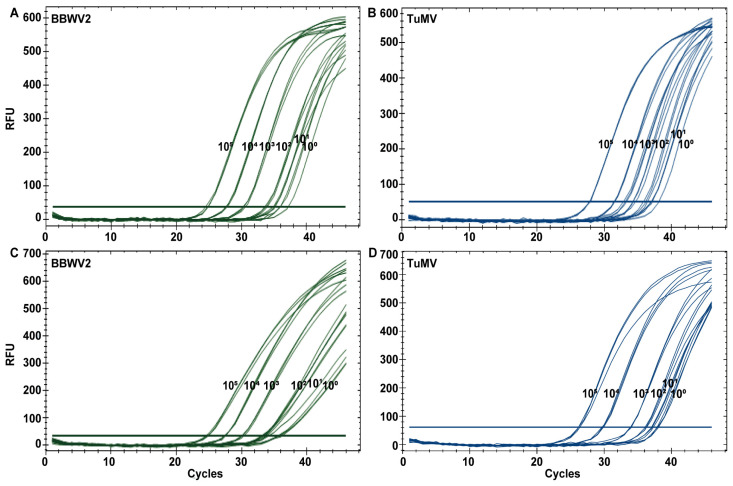
The evaluation for the initial detection range of singleplex (**A**,**B**) and duplex (**C**,**D**) TaqMan qPCR by using virus standard plasmid with a range of 10^5^ to 10^0^ copies/μL.

To preliminary evaluate the sensitivity and the initial detection range of singleplex and duplex TaqMan qPCR, the plasmid standards of TuMV and BBWV2 with a concentration of 10^0^–10^5^ copies/μL were tested using optimised TaqMan qPCR (Figure 2). A Cq value of ≤35 was employed as the Cq value determining the lower detectable limit of qPCR for the TuMV and BBWV2. The results demonstrate that, for both singleplex and duplex TaqMan qPCR assays, the lower detection limits are 2.38 × 10^1^ copies/μL for BBWV2 and 1.12 × 10^3^ copies/μL for TuMV, indicating consistent sensitivity across singleplex and duplex assays (Figure 2).

**Figure 3:**
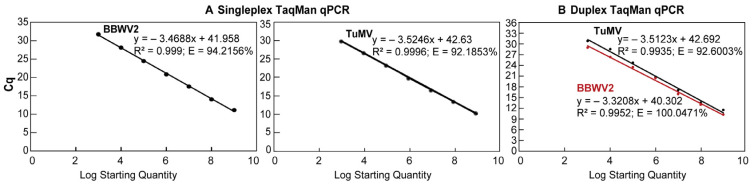
The standard curve of singleplex and duplex TaqMan qPCR. The standard curve for TaqMan qPCR, used for the singleplex (**A**) and duplex detection (**B**) of the BBWV2 or TuMV virus, was identified using the concentrations of virus standard plasmid ranging from 10^9^ to 10^3^. The linear equations between Cq values and virus concentration were obtained by utilising the qPCR Cq values of two viruses as the independent variable (y) and the corresponding plasmid concentration (Log x) as the dependent variable.

Following the initial sensitivity analysis of TaqMan qPCR utilising a dilution series from 10⁵ to 10⁰, it was established that the limit of detection for both viruses was 10^3^ copies, with each dilution point yielding stable Cq values. Accordingly, plasmid concentrations ranging from 10^3^ to 10^9^ were chosen to construct standard curves for qPCR to ensure the accurate quantification of the two viruses. In a singleplex TaqMan qPCR assay for the BBWV2 or TuMV virus, a standard curve for BBWV2 was indicated by the equation y = −3.4688x + 41.958, with an amplification efficiency of 94.2156% and a correlation coefficient (R^2^) of 0.999 (Figure 3A). The standard curve for TuMV was indicated by the equation y = −3.5246x + 42.63, with an amplification efficiency of 92.1853% (E) and a correlation coefficient of 0.999 (R^2^) (Figure 3A). In the duplex TaqMan qPCR assay, the standard curve of BBWV2 was identified as y = −3.3208x + 40.302, exhibiting a correlation coefficient (R^2^) of 0.9952 and an amplification efficiency (E) of 100.0471% (Figure 3B). The standard curve for TuMV is indicated by the equation y = −3.5123x + 42.692, exhibiting a correlation coefficient (R^2^) of 0.9935 and an amplification efficiency (E) of 92.6003% (Figure 3B).

### 3.3. Sensitivity and Reproducibility of Singleplex and Duplex TaqMan qPCR

The results of the probit analysis indicated that 95% LOD of TuMV and BBWV in singleplex qPCR was 734 and 20 copies/μL, respectively; the LOD of TuMV and BBWV in duplex qPCR was found to be 945 copies/μL and 47 copies/μL, respectively. Subsequently, the reproducibility of singleplex and duplex TaqMan qPCR was evaluated. The standard plasmid of TuMV and BBWV2, with a concentration of 10⁴ to 10⁶ copies/μL, was employed as a template in qPCR. The Cq values were recorded at different plasmid concentrations. The results indicated that the variation coefficients of the Cq values in the intra-group and inter-group reproducibility tests for singleplex BBWV2 qPCR ranged from 0.25% to 0.40% and from 0.26% to 1.00%, respectively (Table 3). The variation coefficient of the Cq value was observed to be between 0.54% and 1.04% in intra-group reproducibility tests and between 0.38 and 1.09% in inter-group reproducibility tests for singleplex TuMV qPCR (Table 3). In the duplex TaqMan qPCR assay, the variation coefficient of BBWV2 Cq in intra-group and inter-group reproducibility tests were observed to range from 0.41% to 1.02% and from 0.48% to 0.62%, respectively (Table 3). Additionally, the variation coefficient of TuMV Cq value in the intra-group and inter-group reproducibility analysis ranged from 0.42% to 0.86% and from 0.16% to 0.71%, respectively (Table 3). These findings demonstrate that the inter-group and intra-group coefficients of variation for singleplex and duplex virus qPCR detection are less than 1.2%. Consequently, the singleplex and duplex virus detection established in this study exhibit excellent reproducibility.

### 3.4. The Use of Duplex TaqMan qPCR for the Investigation of the Levels of Viruses Present in P. heterophylla in Major Production Areas

**Figure 4:**
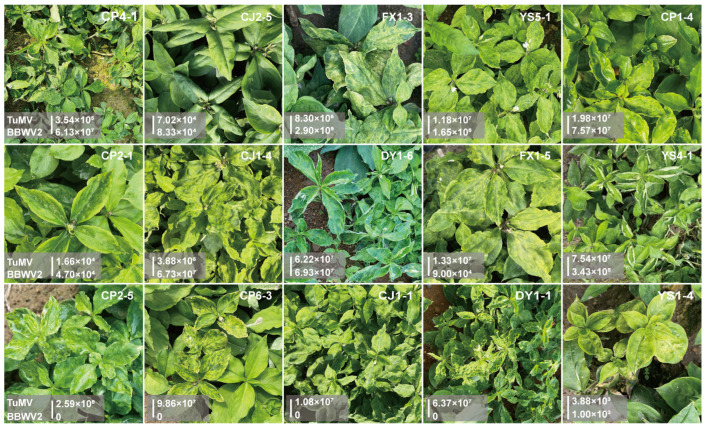
The symptoms of the viral disease of *P. heterophylla* for partial samples from the main cultivation region of *P. heterophylla*. The figure illustrates the infection levels of the TuMV and BBWV2 viruses, as determined via duplex TaqMan qPCR. Detailed infection level data across the various samples can be found in Table S4, which provides a summary of the infection levels in *P. heterophylla* at 60 sampling sites. It should be noted that the present study has only identified *P. heterophylla* TuMV and BBWV2. However, *P. heterophylla* has previously been shown to harbour a variety of viruses and their subtypes. Consequently, the virus symptoms displayed in the figure may not wholly be attributable to the presence of these two viruses. It is more probable that the observed disease results from a combination of multiple viral infections.

To determine the prevalence of TuMV and BBWV2 viruses in *P. heterophylla* grown in the field, *P. heterophylla* plants from 60 sampling sites were collected from the main cultivated areas of *P. heterophylla*. Duplex TaqMan qPCR was used to determine the infection levels of the two viruses in *P. heterophylla* samples. The results demonstrated that *P. heterophylla* was effectively detected as being infected with TuMV at 58 out of the 60 sampling sites, indicating an infection rate of 96% (Figure 4, Table S4). Of the samples infected with TuMV, *P. heterophylla* at 9 sampling sites had an infection level above 10^8^ copies/μL, 40 sampling sites had an infection level between 10^5^ copies/μL and 10^7^ copies/μL, and 9 sampling sites had an infection level below 10^4^ copies/μL (Figure 4, Table S4). *P. heterophylla* at 42 sampling sites were found to be infected with BBWV2, representing an infection rate of 70%. Of these, *P. heterophylla* at 13 sampling sites had an infection load greater than 10^8^ copies/μL, 14 sampling sites had a level between 10^5^ copies/μL and 10^7^ copies/μL, and 15 sampling sites had a level less than 10^4^ copies/μL. In addition, *P. heterophylla* at 40 sampling sites were found to be infected with both TuMV and BBWV2, with 34 samples having viral levels greater than 10⁴ copies/μL (Figure 4, Table S4). The above results showed that *P. heterophylla* over 96% in sampling areas were infected with one of the TuMV and BBWV2 viruses, with TuMV having the highest infection rate, followed by BBWV2. Furthermore, the distribution of virus levels in co-infected samples indicates that TuMV has a higher prevalence than BBWV2.

Notably, of the two viruses, TuMV virus exhibits a relatively limited degree of variation, and its hydrolysis probes and primers are capable of detecting the currently identified three TuMV subtypes including TuMV-ZR (OK323197.1), TuMV-TFJ (OR178748), and TuMV-AHBL(MW017473.1). The BBWV2 virus exhibits considerable genetic diversity, with six distinct subtypes currently identified in *P. heterophylla* from disparate geographical locations [12]. In this study, the probe and primer for BBWV2 were designed primarily based on the previous identification of BBWV2 [16,17], which belongs to the BBWV2 XC subtype identified by Li et al. [12]. Given the considerable variation observed in BBWV2, a comparison was made between the binding region information of the probe and primer on the BBWV2 subtype genome. It was found that the current probe and primer could only detect two of the six BBWV2 subtypes including BBWV2-XC1 (ON241334) and BBWV2-ZR-GX2 (ON241332). It can be concluded that the BBWV2 viruses identified in the above *P. heterophylla* in the field were only two of the six BBWV2 subtypes. Further investigation of the other BBWV2 subtypes that have infected the *P. heterophylla* plants is necessary for future studies.

### 3.5. Monitoring Virus Levels During the Process of BBWV2 Infecting P. heterophylla Through Singleplex TaqMan qPCR

**Figure 5:**
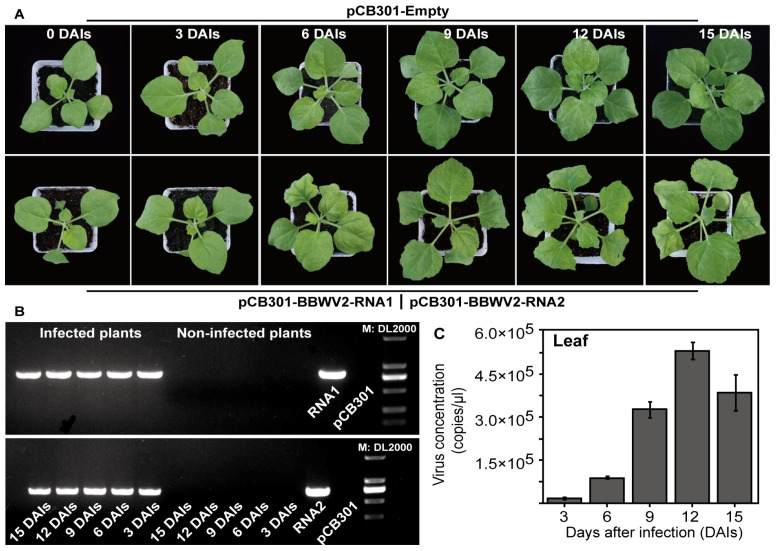
The phenotypic characteristics and BBWV2 levels of *N. benthamiana* following infection with BBWV2 infectious clones. The external characteristics *N. benthamiana* following infection with BBWV2 infectious clones were observed (**A**). The presence of the BBWV2 infectious clone in *N. benthamiana* was determined through the use of PCR methods (**B**). The singleplex qPCR was employed to detect BBWV2 levels during its process of infecting *N. benthamiana* (**C**).

To further elucidate the utilisation of TaqMan qPCR in the investigation of the interaction between a virus and *P. heterophylla*, an infectious clone of *P. heterophylla* BBWV2 was introduced into both *P. heterophylla* and *N. benthamiana* through the infiltration technique. The levels of BBWV2 in both plants were monitored using singleplex TaqMan qPCR. As a consequence of the prolonged infectious stages following BBWV2 infiltration, the leaves of *N. benthamiana* exhibited a curling effect and a gradual loss of chlorophyll (Figure 5A). Furthermore, the infection states of BBWV2 in *N. benthamiana* were confirmed through RT-PCR methods (Figure 5B). The findings indicated that the 823 bp BBWV2-RNA1- and 727 bp BBWV2-RNA2-fragments could be distinctly amplified in *N. benthamiana* plants on different days after infection (DAIs), in alignment with the anticipated size (Figure 5B). These results thus confirm that BBWV2 can successfully infect *N. benthamiana*. The TaqMan qPCR results demonstrated a gradual increase in BBWV2 levels in *N. benthamiana* leaves after viral infection, reaching a peak at 12 DAIs and subsequently declining by 15 DAIs (Figure 5C).

**Figure 6:**
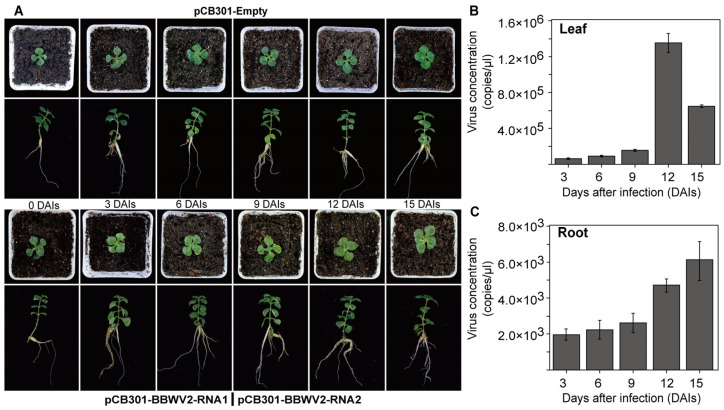
The phenotypic characteristics and BBWV2 levels of *P. heterophylla* following the infection with BBWV2 infections clones. The phenotypic characteristics of *P. heterophylla* at varying stages after injection with BBWV2 infectious clones were observed (**A**). BBWV2 levels in the leaves and roots of these *P. heterophylla* were identified through singleplex TaqMan qPCR for BBWV2 detection (**B**,**C**).

The analysis of BBWV2 infection in *P. heterophylla* revealed that the primary symptoms were observed on the leaves, characterised by yellowing and slight distortion (Figure 6A). To confirm the presence of BBWV2 in *P. heterophylla*, RT-PCR methods were employed. The results demonstrated that the RNA1 and RNA2 fragments of the BBWV2 virus were successfully amplified in the infected *P. heterophylla*, exhibiting a size consistent with the expected range (Figure S5). The results of the qPCR analysis indicated that the levels of BBWV2 peaked in the aboveground portions at 12 DAIs, with a slight decline at 15 DAIs (Figure 6B). In roots, BBWV2 levels were typically lower but increased gradually with infection time, indicating a potential spread from leaves to roots (Figure 6C). A comparative analysis of the changes in BBWV2 levels in the two plants revealed that BBWV2 levels initially increased and subsequently decreased with the prolongation of infection time, particularly at 15 DAIs.

## 4. Discussion

Despite the identification of the primary pathogens responsible for *P. heterophylla* viral disease, the precise infection levels remain uncertain, hindering the implementation of effective control measures. It is of utmost importance to promptly monitor the status of virus infection, assess the severity of infection, and employ effective control technologies in the production of *P. heterophylla*. TaqMan qPCR technology allows for the precise quantification of infection levels of diverse viruses in instances of co-infection [19,20,21,22]. This provides a crucial point of reference for the development of prevention and control strategies for co-infecting viruses. The development of a TaqMan qPCR detection method is therefore essential for the control of *P. heterophylla* virus infection.

The construction of a qPCR system for the detection of viral diseases in *P. heterophylla* requires the complete genome and corresponding viral plasmid to be available. Currently, numerous viruses have been identified across various cultivation regions of *P. heterophylla* [12,17]. Of these, TuMV and BBWV2 are considered the primary agents causing the viral disease that affects *P. heterophylla*. A previous study has successfully identified the complete genomes and corresponding cloning plasmids of the two core viruses, TuMV and BBWV, thereby paving the way for the development of a qPCR method for viral detection. The design of hydrolysis probes and primers is of pivotal importance in enhancing the analytical sensitivity, specificity, and efficiency of viral detection [22,23,24,25]. However, challenges for viral detection emerge due to sequence similarities among viruses, which can result in non-specific amplification. Additionally, the viral RNA polymerase’s inability to correct errors during viral replication can lead to the emergence of a multitude of viral subtypes [26]. All of these factors have the potential to compromise the accuracy and validity of the qPCR detection. To address these challenges, this study analysed the conservation of the whole viral genome to design hydrolysis probes and primers that would specifically target conserved regions. On this basis, the qPCR parameters, including annealing temperature, primer concentration, and hydrolysis probe concentration, were optimised in detail for the effective detection of singleplex or duplex viruses. Among the three parameters, the annealing temperature between singleplex and duplex viruses was found to be consistent, while the other two parameters exhibited slight differences, indicating that there was minimal interference between primers and hydrolysis probes from the TuMV and BBWV2 virus. Moreover, the sensitivity and reproducibility of the determination of both singleplex and duplex virus qPCR in subsequent assays revealed that the primers and hydrolysis probes designed by this study exhibited effective detection results for the two *P. heterophylla* viruses.

The sensitivity of qPCR detection is a crucial parameter for assessing the efficacy of this technique. In the sensitivity assay, the minimum detection limits (95% LOD) for the singleplex qPCR of TuMV and BBWV2 were higher than to those of the duplex virus qPCR, respectively. The multiplex qPCR comprises multiple pairs of primers and hydrolysis probes, with interactions among them being more complex than those observed in a singleplex qPCR [25,27,28,29,30]. The findings further suggested that the primers and probes used together in a duplex qPCR to detect the TuMV and BBWV2 exhibited minimal interference with one another. A test of reproducibility was conducted on singleplex and duplex qPCR in order to ascertain the coefficient of variation for both intra- and inter-assay variability. The resulting values were all below 1.2%, indicating that the developed singleplex and duplex virus TaqMan qPCR method is highly reproducible. Accordingly, this approach can be employed for the quantitative detection of *P. heterophylla* viral disease, thereby facilitating a technical means for the rapid identification of the *P. heterophylla* viral disease.

One of the most crucial applications of the TaqMan qPCR method is the diagnosis of viral pathogens. In this study, two viruses affecting *P. heterophylla* in the main cultivation regions were thus detected by the TaqMan qPCR method. The results demonstrated that the majority of *P. heterophylla* plants in the field exhibited evidence of infection with at least one of the two viruses, with prevalence reaching 90%. This finding is largely following the existing detection outcomes obtained through RT-PCR [1,8,9,10]. A higher infection rate and greater abundance of TuMV were identified in comparison to that of BBWV2. Of the two viruses, BBWV2 is rarely observed as a single infection, with the majority of cases involving co-infection with TuMV. The observed result can be attributed primarily to the distinct infection mechanisms of the two viruses. Several proteins in TuMV, including VPg, Hc-Pro, and P1, have been characterised as having the function of antiviral silencing suppressors, thereby conferring viral resistance to elimination by the host plant [31,32,33]. In contrast, BBWV2 is devoid of robust antiviral silencing suppressors, rendering it susceptible to the RNA silencing system of the plant [34]. A comparative analysis of the symptoms and virus levels revealed a notable similarity in the observed symptoms, yet considerable divergence in the virus levels exists. In some instances, the virus levels appeared to be comparable, yet the symptoms exhibited notable discrepancies. The observed discrepancy can be attributed to some factors, primarily related to the distinct cultivation management practices applied to *P. heterophylla*, as well as the inherent genetic diversity among different *P. heterophylla* genotypes. Moreover, the internal virus presence of the planted material and the algebra of their reproductive material are of crucial significance to this phenomenon [34]. Moreover, the specific virus strain that infects *P. heterophylla* also plays a role in this phenomenon. It should be noted that the present study was designed exclusively to detect and quantify TuMV and BBWV2. The potential presence of other viruses was not investigated. However, it is plausible that undetected viruses or viral subtypes may also play a crucial role in the development of the observed symptomology. This also demonstrates that the symptoms caused by the viruses in the field are affected by multiple factors.

Moreover, TaqMan qPCR can be utilised to determine the accumulation levels of viruses in host plants and to assess the extent of virus-induced damage to plants. This study employed BBWV2 infection clones as infection materials and analysed the accumulation and transmission of the viruses in *P. heterophylla* under controlled conditions to gain preliminary insight into the diffusion dynamics of viruses in *P. heterophylla*. The results demonstrated that the BBWV2 virus exhibited a rapid proliferation following infection, reaching more than 10^6^ copies/μL in leaves at 12 DAIs, and subsequently declining. These findings are following the results of the infection process of pepper BBWV2 infectious clones [35]. The observed gradual decline in BBWV2 infection levels within *P. heterophylla* plants may be attributed to the absence of the viral suppression of RNA silencing in BBWV2. This results in the elimination of the viral genome by the plant’s antiviral immune system during infection, thereby preventing the further spread of the virus [35]. The aforementioned field investigation of the infection status of TuMV and BBWV2 also demonstrated that BBWV2 rarely infects *P. heterophylla* independently. Additionally, several pivotal factors may potentially impact the accrual of viral infectious clones in plant systems. Firstly, the present study introduces BBWV2 infectious clones into plants via the direct injection of *A. tumefaciens*. It is essential to consider the composition of the infection solution, which contains the infectious clone, and the OD values of the solution [36]. Secondly, the efficiency of *A. tumefaciens* infection and the transcription rate of viral genome RNA also exert an influence on the infection of agrobacteria-mediated viral infection clones [37,38]. Lastly, the infecting organism influences the accumulation of infectious viral clones [36]. The present study selected virus-free tissue culture seedlings of *P. heterophylla* thickened leaves, which may affect the invasion levels of *A. tumefaciens* [17]. Although a single viral infection does not fully reflect the distribution and transmission patterns of the virus in natural conditions, it does reflect the infection process of the virus in *P. heterophylla*. This provides a crucial point of reference for determining the optimal time for the implementation of virus control measures.

## 5. Conclusions

In this study, the *P. heterophylla* BBWV2 cloning and infectious cloning vectors were constructed. This was combined with the TuMV cloning vector, and the TaqMan qPCR-based singleplex and duplex virus detection methods were optimised for the separate and simultaneous quantitative detection of two core *P. heterophylla* viruses, BBWV2 and TuMV. Concurrently, the prevalence of the two core viruses in the primary production region of *P. heterophylla* and the infection states of *P. heterophylla* viruses in disparate cultivation sites were determined through the utilisation of duplex virus qPCR. Furthermore, this study examines the alterations in the concentration of the BBWV2 infectious clone throughout its infection of *P. heterophylla*, employing singleplex qPCR. In conclusion, the singleplex and duplex viral qPCR detection methods for *P. heterophylla* BBWV2 and TuMV established in this study are valuable for diagnosing the viral infection status and developing effective control strategies. Furthermore, this study provides essential technical support for the establishment of safe and sustainable production systems for *P. heterophylla*, including the detection of seedling viruses, the assessment of detoxification efficiency, and the monitoring of virus infection in *P. heterophylla*.

## Figures and Tables

**Table 1:** Optimisation of the annealing temperature for a duplex TaqMan qPCR assay employed for the simultaneous detection of the TuMV and BBWV2 viruses.

Annealing Temperature (°C)	Standard Plasmid	Cq (Mean ± SD)	Standard Plasmid	Cq (Mean ± SD)
56	BBWV2	10.97 ± 0.225	TuMV	11.81 ± 0.076
58	BBWV2	10.54 ± 0.240	TuMV	11.14 ± 0.131
60	BBWV2	11.60 ± 0.128	TuMV	11.86 ± 0.071
62	BBWV2	11.07 ± 0.273	TuMV	10.92 ± 0.375
64	BBWV2	10.72 ± 0.267	TuMV	10.78 ± 0.555

**Table 2:** The Cq values (Mean ± SD) for qPCR under the specified primer and probe concentrations required for the optimisation of a duplex TaqMan qPCR assay for the simultaneous detection of the TuMV and BBWV2 viruses.

Viruses	Probe Concentration (μM)	Primer Concentration (μM)			
		0.1	0.2	0.3	0.4
BBWV2	0.05	9.07 ± 0.044	10.14 ± 0.067	10.34 ± 0.346	10.02 ± 0.072
BBWV2	0.10	8.71 ± 0.150	9.99 ± 0.325	10.15 ± 0.139	9.53 ± 0.197
BBWV2	0.15	8.64 ± 0.137	9.84 ± 0.014	9.87 ± 0.088	9.59 ± 0.035
BBWV2	0.20	8.37 ± 0.027	10.01 ± 0.04	10.01 ± 0.031	11.00 ± 0.107
BBWV2	0.25	8.12 ± 0.145	9.92 ± 0.051	9.76 ± 0.108	9.48 ± 0.061
TuMV	0.05	9.64 ± 0.296	11.19 ± 0.045	11.47 ± 0.094	11.33 ± 0.037
TuMV	0.10	10.25 ± 0.038	11.24 ± 0.097	11.02 ± 0.048	11.04 ± 0.014
TuMV	0.15	9.56 ± 0.436	10.95 ± 0.134	10.71 ± 0.473	11.00 ± 0.133
TuMV	0.20	9.63 ± 0.142	11.19 ± 0.086	11.12 ± 0.096	10.67 ± 0.035
TuMV	0.25	10.21 ± 0.346	11.07 ± 0.053	11.18 ± 0.016	10.94 ± 0.083

**Table 3:** Reproducibility assay for a singleplex and duplex TaqMan qPCR for the individual and simultaneous detection of the TuMV and BWV2 viruses.

Plasmid Standards	Concentration (Copies/μL)	Intra-Assay			Inter-Assay		
		Average Cq	SD	CV (%)	Average Cq	SD	CV (%)
Singleplex qPCR							
BBWV2	2.38 × 10^6^	21.57	0.09	0.40	20.85	0.06	0.26
BBWV2	2.38 × 10^5^	24.46	0.06	0.25	24.49	0.07	0.29
BBWV2	2.38 × 10^4^	27.42	0.11	0.40	28.16	0.28	1.00
TuMV	1.12 × 10^6^	20.8	0.22	1.04	21.28	0.09	0.44
TuMV	1.12 × 10^5^	25.05	0.14	0.54	25.04	0.09	0.38
TuMV	1.12 × 10^4^	29.03	0.22	0.77	28.87	0.31	1.09
Duplex qPCR							
BBWV2	2.38 × 10^6^	21.01	0.09	0.41	20.98	0.1	0.48
BBWV2	2.38 × 10^5^	24.16	0.18	0.73	24.1	0.12	0.52
BBWV2	2.38 × 10^4^	27.17	0.28	1.02	27.11	0.17	0.62
TuMV	1.12 × 10^6^	23.89	0.1	0.42	23.83	0.04	0.16
TuMV	1.12 × 10^5^	25.57	0.22	0.86	25.46	0.18	0.71
TuMV	1.12 × 10^4^	29.62	0.21	0.70	29.56	0.09	0.31

## Data Availability

The original contributions presented in this study are included in the article and Supplementary Materials. Further inquiries can be directed to the corresponding authors.

## Supplementary Materials

The following supporting information can be downloaded at: https://www.mdpi.com/article/10.3390/microorganisms12122663/s1, Figure S1: The validation of the presence of pSMART-E-BBWV2-RNA1 plasmid in *Escherichia coli* (*E. coli*) DH10B by PCR method; Figure S2: The validation of the presence of pCB301-BBWV2-RNA1 plasmid in *E. coli* DH10B; Figure S3: The validation of the presence of pSMART-E-BBWV2-RNA2 plasmid in *E. coli* DH10B by PCR method; Figure S4: The validation of the presence of pCB301-BBWV2-RNA2 in *E. coli* DH10B by PCR method; Figure S5: The verification of the infection states of BBWV2 in *P. heterophylla* infected by BBWV2 infectious clone through RT-PCR method; Table S1: List of primers used in this study; Table S2: Optimisation of the annealing temperature for a singleplex TaqMan qPCR used for the individual detection of TuMV or BBWV2 virus; Table S3: Determination of primer and probe concentration in singleplex TaqMan qPCR for the individual detection of TuMV or BBWV2 virus; Table S4: A comparative analysis of the levels of TuMV and BBWV2 viruses in *P. heterophylla* from different sampling sites of mainly cultivated regions.
