# Supplementary material for: Development of a TaqMan qPCR for the Simultaneous Detection of the TuMV and BBWV2 Viruses Responsible for the Viral Disease in Pseudostellaria heterophylla
Source: Microorganisms. 2024 Dec 22;12(12):2663. doi: 10.3390/microorganisms12122663 (PMC11728560; doi:10.3390/microorganisms12122663)

Supplementary Figures includes 5 figures

Figure S1. The validation of the presence of pSMART-E-BBWV2-RNA1 plasmid in *Escherichia coli* (*E. coli*) DH10B by PCR method. Two pairs of primers, BBWV2-RNA1-ClonP1 and BBWV2-RNA1-ClonP2 (Table S1), which are specific to the BBWV2 virus, were utilised to amplify the BBWV2-RNA1-P1 and BBWV2-RNA1-P2 regions from BBWV2-RNA1, respectively. Lane 1-5 and Lane 6-10 illustrate the amplification of the BBWV2-RNA1-P1 and BBWV2-RNA1-P2 fragments, respectively, from five randomly selected *E. coli* clones.

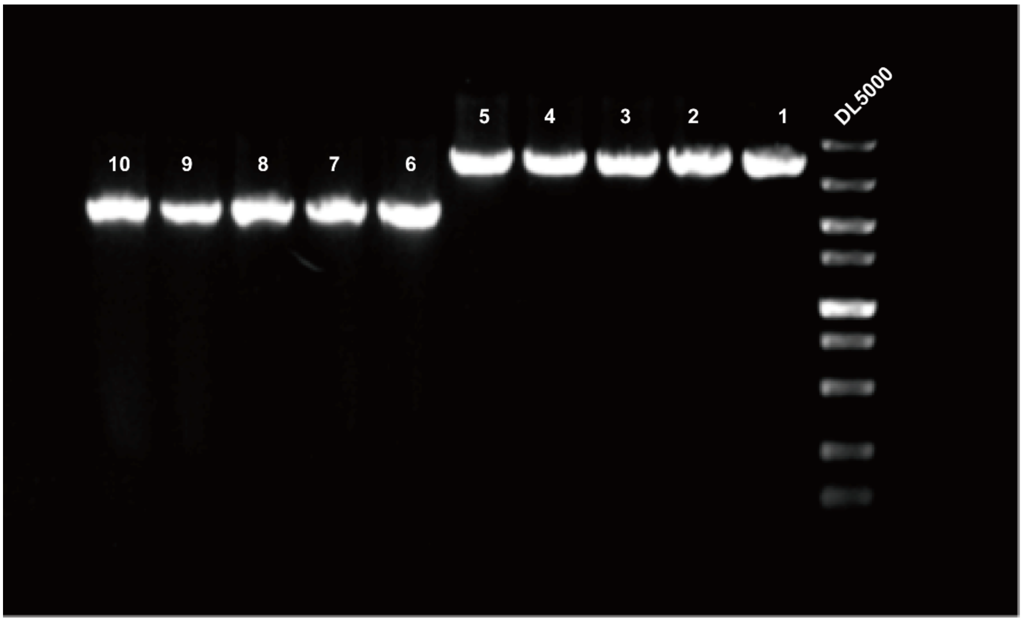

Figure S2. The validation of the presence of pCB301-BBWV2-RNA1 plasmid in *E. coli* DH10B. This process employed the BBWV2-RNA1-ExP1 and BBWV2-RNA1-ExP2 primers (Table S1) to amplify the pCB301-BBWV2-RNA1-P1 and pCB301-BBWV2-RNA1-P2 regions, respectively, from pSMART-E-BBWV2-RNA1 plasmid. Lane 1-5 and Lane 6-10 demonstrate the amplification of both the BBWV2-RNA1-P1 and BBWV2-RNA1-P2 fragments, respectively, from five randomly selected *E. coli* clones.

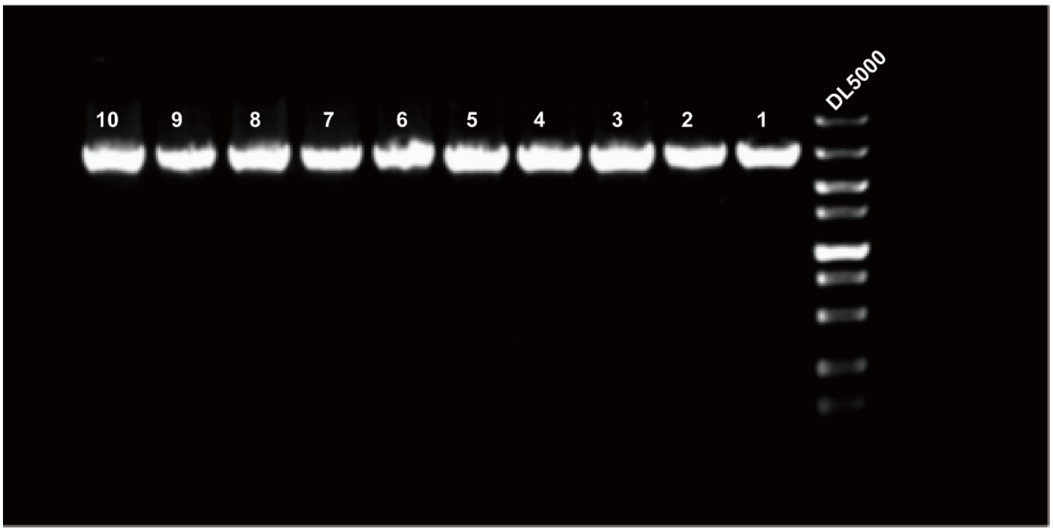

Figure S3. The validation of the presence of pSMART-E-BBWV2-RNA2 plasmid in *E. coli* DH10B by PCR method, utilising BBWV2-ClonRNA2 primers (Table S1) for the amplification of BBWV2-RNA2. Lane 1-4 demonstrates the amplification of BBWV2-RNA2 from four randomly selected *E. coli* clones.

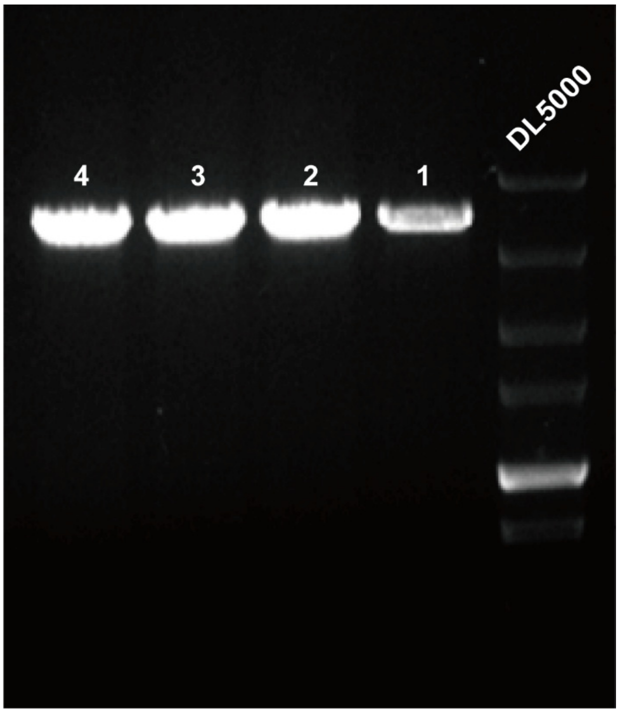

Figure S4. The validation of the presence of pCB301-BBWV2-RNA2 in *E. coli* DH10B by PCR method. This is achieved by the utilisation of BBWV2-ExRNA2 primers (Table S1) for the amplification of the BBWV2-RNA2 from pSMART-E-BBWV2-RNA2 plasmid. Lane 1-4 illustrates the amplification of the BBWV2-RNA2 from four randomly selected *E. coli* clones.

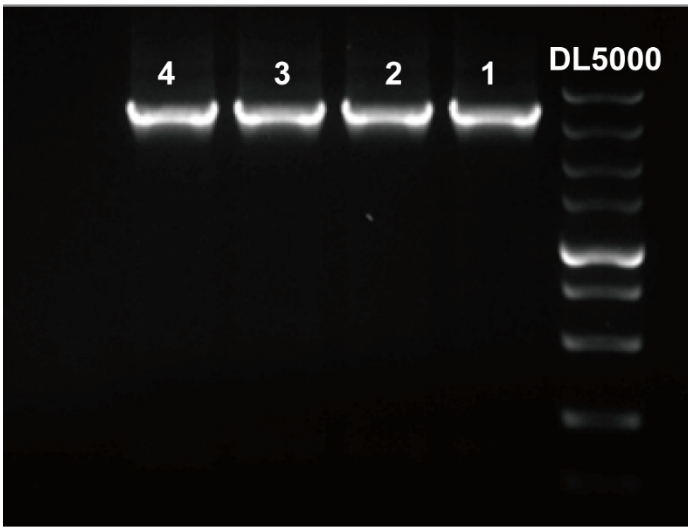

Figure S5. The verification of the infection states of BBWV2 in *P. heterophylla* infected by BBWV2 infectious clone through RT-PCR method. The specific fragments from BBWV2-RNA1 (A) and BBWV2-RNA2 (B) were amplified in *P. heterophylla* infected by BBWV2 infectious clone using the specific primers (Table S1) BBWV2-RNA1-Dect and BBWV2-RNA2-Dect, respectively.

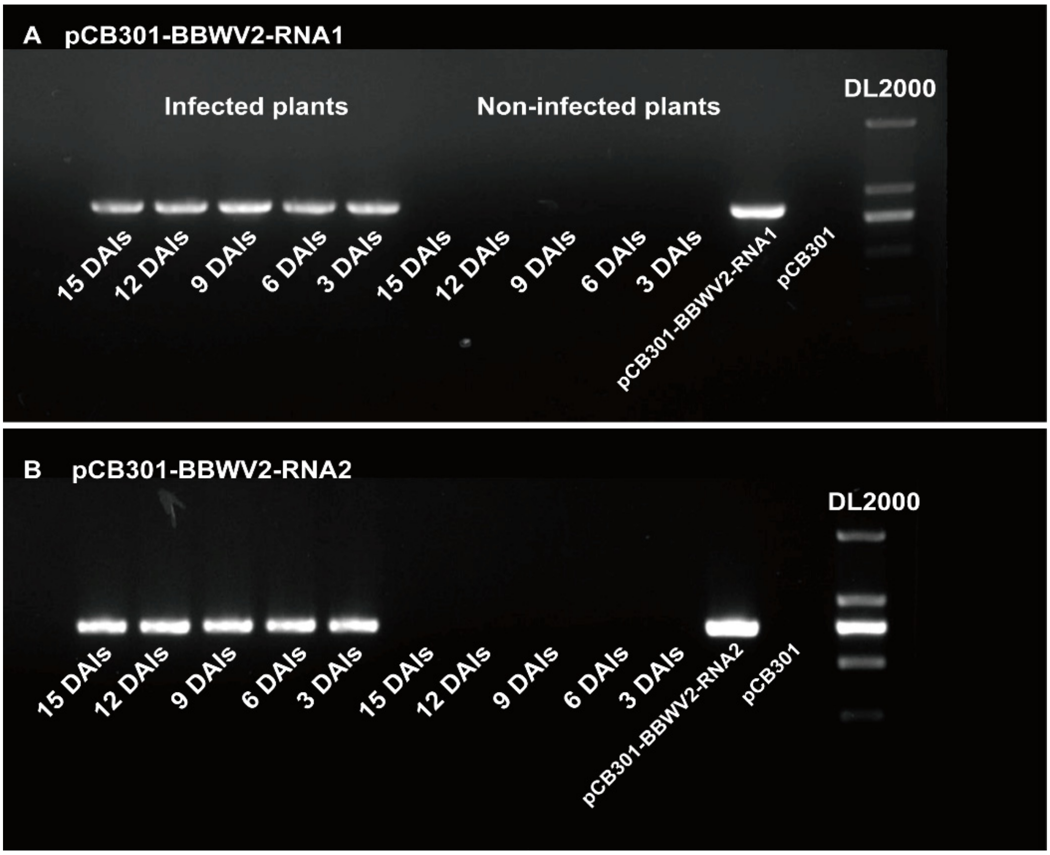

Supplement: Supplementary file 1 [file microorganisms-12-02663-s001.zip › Supplementary Figures.pdf]
